# Endoscopic Versus Microscopic Transsphenoidal Surgery in the Treatment of Pituitary Adenoma: A Systematic Review

**DOI:** 10.7759/cureus.107051

**Published:** 2026-04-14

**Authors:** Diego Santillán Alcántar, Flor Belén Villalobos Villalobos, Alejandro Antonio Merazo Valle, Axel Yahir Rodríguez Muñoz, Sergio Yared Gutierrez Chavez, Alejandro Gutierrez, Jose R Flores Valdés, Axel Otniel Romero Flores, Margith de los Angeles Izaguirre Arostegui, Ramiro Daniel Montero Coutiño, Reynaldo Arce Martinez, Vicky Basurto Osorio, Juan Blanco González, Jaqueline Castillo, Orlando Tortolero Barrón

**Affiliations:** 1 General Surgery, Instituto Mexicano del Seguro Social, Hospital General Regional No. 46, Guadalajara, MEX; 2 General Medicine, Universidad Autónoma de Coahuila, Torreón, MEX; 3 General Medicine, Universidad de El Salvador, San Salvador, SLV; 4 General Medicine, Universidad de Guanajuato, León, MEX; 5 General Medicine, Escuela Libre de Homeopatía de México, Mexico City, MEX; 6 General Medicine, Universidad del Valle de México, Zapopan, MEX; 7 General Practice, Oncology Consultants PA, Houston, USA; 8 General Medicine, Campus Universitario Siglo XXI, Zinacantepec, MEX; 9 General Medicine, Universidad Nacional Autónoma de Nicaragua, Managua, NIC; 10 General Medicine, Universidad Autónoma de Chiapas, Tuxtla Gutiérrez, MEX; 11 General Medicine, Universidad de la Salud de la Ciudad de México, Mexico City, MEX; 12 General Medicine, Universidad Cristóbal Colón, Veracruz, MEX; 13 General Medicine, Benemérita Universidad Autónoma de Puebla, Puebla, MEX; 14 General Practice, Universidad Autónoma de Guadalajara, Zapopan, MEX; 15 Neurological Surgery, Instituto Mexicano del Seguro Social, Hospital General de Zona No. 1, Tepic, MEX

**Keywords:** endoscopic, microscopic, pituitary adenoma, pituitary gland, transsphenoidal

## Abstract

Pituitary adenomas are the most prevalent pituitary pathology and originate from the neoplastic proliferation of anterior pituitary lobe cell lineages. Two primary surgical approaches are employed: microscopic transsphenoidal surgery (MTS) and endoscopic transsphenoidal surgery (ETS). Both approaches aim to optimize tumor resection while reducing the risk of complications. The objective of this review is to compare the outcomes of ETS and MTS in the treatment of pituitary adenomas.

Studies from 1974 to March 20, 2026, from PubMed, ScienceDirect, and Cochrane databases were included. The inclusion criteria were patients diagnosed with pituitary adenomas and patients aged ≥18 years.

The systematic review includes a total of 22 articles with a total of 4390 participants, of which 2413 underwent ETS and 1973 underwent MTS. The findings suggest that gross total resection (GTR) was more frequently reported in patients undergoing the endoscopic technique; however, considerable variability exists across studies. Postoperative complication rates were variable and showed substantial overlap between endoscopic and microscopic techniques. Although the endoscopic technique may provide some benefits, overall safety and recovery outcomes seem to be affected more by study features and the surgeon's experience than by the surgical method itself.

The comparison between both surgical techniques suggests potential advantages for the endoscopic approach in terms of GTR; however, clinical outcomes, including complications, recovery, and endocrine function, are comparable between techniques and appear to be influenced more by surgical expertise, tumor characteristics, and study design than by the surgical approach alone.

## Introduction and background

The pituitary gland is located within the sella turcica of the sphenoid bone, inferior to the optic chiasm and in close relation to the cavernous sinuses, and is connected to the hypothalamus via the pituitary stalk. The adenohypophysis, which comes from Rathke's pouch, and the neurohypophysis, which comes from neural ectoderm, are its two embryologically separate parts [1].

Pituitary adenomas are the most common type of pituitary disorder and include neoplasms of the anterior pituitary lobe cell lineage. They can be classified based on their primary cellular origin and the type of hormone secreted. If they do not secrete sufficient hormones to be detected in the blood or cause any clinical manifestations, they are considered non-functioning. Based on this, pituitary adenomas include prolactinomas, which are the most frequent functioning tumors, as well as somatotropinomas, corticotropinomas, thyrotropinomas, and gonadotropinomas, while most non-functioning adenomas arise from gonadotroph cell lineage [2].

Pituitary adenomas can also be classified based on size. Macroadenomas measure ≥10 mm, whereas microadenomas measure <10 mm, with microadenomas being more common [3-5]. Hormonal hypersecretion may lead to distinct endocrine syndromes, such as Cushing's disease in corticotropinomas and acromegaly in somatotropinomas [6]. It is estimated that approximately 10% of the population will develop a pituitary adenoma; however, most remain asymptomatic and undiagnosed throughout life. The tumor accounts for 15% of intracranial neoplasms, making it one of the most common brain tumors [7,8]. The prevalence of clinically evident pituitary adenoma in the general population is between one in 1100 people [4].

The transsphenoidal approach has become the gold standard for the surgical treatment of pituitary adenomas, offering a minimally invasive route through the sphenoid sinus to access the tumor [9]. Two primary techniques are employed: microscopic transsphenoidal surgery (MTS) and endoscopic transsphenoidal surgery (ETS). The microscopic technique, established as the traditional approach, uses an operating microscope to provide a high-resolution visualization of the surgical field. In contrast, the endoscopic technique employs a rigid endoscope, which enhances visualization with a wider field of view, superior illumination, and better access to complex anatomical regions. Both methods aim to maximize the extent of tumor resection while minimizing complications [6,10].

In the literature, there are studies comparing microscopic with endoscopic transsphenoidal resection in the treatment of pituitary adenomas. However, there remains a lack of consensus on which approach provides superior results across diverse clinical scenarios. The question remains regarding differences in long-term outcomes. Furthermore, variations in complication rates and recovery times across techniques highlight the need for a comprehensive synthesis of the available evidence to guide clinical decision-making [11].

The primary objective of this systematic review is to compare outcomes of MTS and ETS in the treatment of pituitary adenomas. This review evaluates gross total resection (GTR), postoperative complication rates, and recovery times. By synthesizing evidence from clinical studies, this analysis aims to provide clinicians with a clearer understanding of the relative advantages and limitations of each approach, ultimately supporting informed surgical decisions and improving patient outcomes.

## Review

Methods

Search Strategy

This systematic review complies with the guidelines established by the Preferred Reporting Items for Systematic Reviews and Meta-Analyses (PRISMA) 2020 [12,13]. The studies included declared compliance with the ethical standards of the Declaration of Helsinki, with registration officially obtained and validated through the International Prospective Register of Systematic Reviews (PROSPERO) with the ID CRD42025635492 [14].

The literature search was conducted in PubMed, ScienceDirect, and Cochrane databases. The search strategy employed Medical Subject Headings (MeSH) terms and accessible text terms to March 20, 2026. We utilized the following keywords: pituitary adenoma, microscopic transsphenoidal approach, and endoscopic transsphenoidal approach. The search strategy was sent as supplementary material.

Type of Study

The inclusion criteria are studies published in English covering the period from 1974 to March 2026, utilizing methodologies such as randomized controlled trials, cohort studies, or case-control studies that compare the use of ETS with MTS in pituitary adenomas.

The exclusion criteria include case reports, expert opinions, case series, letters to the editor, book chapters, comment publications, and dissertations. Additionally, studies that did not detail their methodology, duplicates, and those where necessary data could not be obtained or where the author did not respond to email inquiries were excluded.

Type of Participants

The inclusion criteria for participants included adult patients (≥18 years) diagnosed with pituitary adenomas of any functional or histological subtype. Both male and female patients of any ethnic background were included.

We excluded studies involving patients younger than 18 years, pregnant women, animal subjects, and tumors other than pituitary adenomas in the sellar region.

Type of Intervention

The inclusion criteria for the intervention group consisted of patients who underwent ETS as the current standard surgical treatment for pituitary adenomas. The control group consists of patients who underwent MTS for the same condition. The exclusion criteria for patients include those treated with a mixed technique or any other approach different from the transsphenoidal method.

Outcomes

In this systematic review, the main objective was to evaluate the degree of tumor resection between the two surgical interventions by classifying it as GTR. This is in line with previous comparative studies where they concluded that the quality of resection is significantly improved after one year in patients who underwent endoscopic surgery, supporting the use of GTR as a relevant primary outcome in comparative surgical studies [15]. Although GTR is frequently used as an endpoint in comparative surgical studies, it is crucial to understand that it is not necessarily the main surgical objective, especially when avoiding endocrine morbidity and maintaining pituitary function are the top priorities.

Secondary outcomes include postoperative complications, including the development of meningitis, sinusitis, epistaxis, diabetes insipidus, cerebrospinal fluid (CSF) leak, and operative time; recovery times, considering the length of hospital stay (LOS) and follow-up months in each study; and the impact on vision, whether there has been an improvement or deterioration.

Selection of Studies

In the study selection process, we used the Rayyan platform (Rayyan Systems Inc., Cambridge, MA) [16]. The initial selection of studies was based on the title and abstract, where two independent reviewers (AORF and AAMV) selected studies according to our inclusion and exclusion criteria. Disagreements were resolved by adding a third reviewer (RDMC). For full-text screening, two independent reviewers (MAIA and AG) selected studies using our inclusion and exclusion criteria. Any discrepancies were resolved by adding a third reviewer (RAM).

Data Collection Process

Data extraction was carried out by three independent reviewers (RDMC, JBG, and FBVV) using predesigned tables that contained the relevant characteristics of the articles, as well as primary and secondary outcomes. For data synthesis, we used a qualitative synthesis guided by a narrative analysis. The findings were described in terms of patterns and trends among the included studies. Tables and figures were used to provide a clear and precise summary of the results. Due to heterogeneity among included studies in terms of design, patient characteristics, tumor classification, and outcome reporting, a formal meta-analysis was not performed.

Assessment of the Risk of Bias of the Included Studies

Two independent reviewers (AORF and AYRM) assessed the risk of bias of the included studies, and any discrepancies were resolved by adding a third reviewer (MAIA) using two scales: the RoB 2 tool [17] for randomized clinical trials and the Newcastle-Ottawa Quality Assessment Scale criteria [18] for cohort studies. The results of the RoB 2 tool are interpreted as low risk, some concerns, or high risk based on the risk of bias. The results of the Newcastle-Ottawa Quality Assessment Scale criteria are interpreted as good quality, fair quality, or poor quality based on the risk of bias.

Results

The review included available information from PubMed, ScienceDirect, and Cochrane databases from 1974 to March 20, 2026.

**Figure 1:**
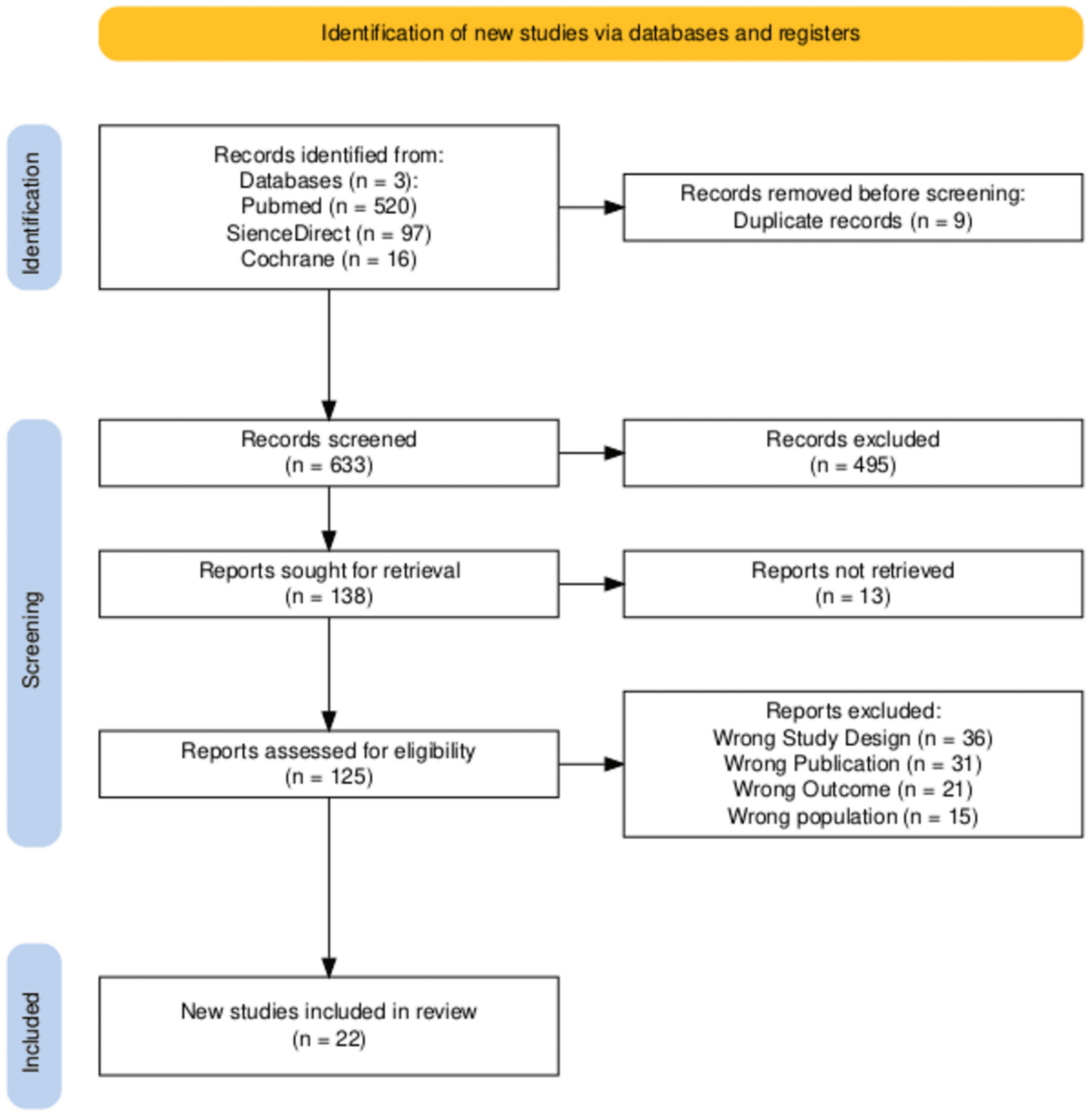
PRISMA flow diagram Note: Initially, 633 articles were screened using the Rayyan platform [16]; 495 were excluded. Afterward, 138 articles were sought for retrieval, of which 13 could not be accessed. A total of 125 full-text articles were assessed for eligibility. Full-text exclusions were due to wrong study design (n = 36), wrong publication type (n = 31), irrelevant outcomes (n = 21), and wrong population (n = 15). Ultimately, 22 studies were included in the final qualitative synthesis. This stepwise illustration provides transparency in our filtering process, which guided the final study selection PRISMA: Preferred Reporting Items for Systematic Reviews and Meta-Analyses

As outlined in the PRISMA chart, a total of 633 studies were retrieved, of which 138 underwent full-text analysis [13]. Ultimately, 22 articles were deemed eligible after removing duplicates, screening titles and abstracts, and excluding non-accessible information, as illustrated in Figure 1.

The systematic review includes a total of 22 articles, of which 19 are cohort studies (86.36%) and three are randomized clinical trials (13.64%). The studies were conducted in various countries, including the USA (22.72%), China (22.72%), Turkey (9.09%), Colombia (4.54%), Canada (4.54%), Iran (4.54%), France (4.54%), Japan (4.54%), Italy (4.54%), Spain (4.54%), and Bulgaria (4.54%). A total of 22 studies were included, comprising 4390 patients, of which 2413 underwent ETS and 1973 underwent MTS. The participants were between 18 and 84 years old. The number of participants per study ranged from 10 to 1023 (Table 1).

**Table 1:** Trial characteristics N/S: not specified

Study	Study Design	Country	Study Dates	Surgical Intervention	Number of Patients	Patient Characteristics			
						Mean Age ± SD (Range)	Sex		Mean Follow-Up ± SD (Range) (Months)
							Female	Male	
Van Gompel et al., 2021 [19]	Retrospective cohort	USA	2014-2019	Endoscopic	261	51.7 ± 0.9	N/S	N/S	25 ± 1
				Microscopic	273	50.3 ± 0.9	N/S	N/S	28 ± 1
Gao et al., 2016 [20]	Retrospective cohort	China	2012-2014	Endoscopic	60	44.6 (19-75)	34	26	7 ± 4.6
				Microscopic	45	48.8 (21-77)	26	19	8 ± 5.2
Fathalla et al., 2015 [21]	Retrospective cohort	Canada	2000-2013	Endoscopic	42	43.2	21	21	56.6
				Microscopic	23	42.1	16	7	56.6
Akbari et al., 2018 [22]	Retrospective cohort	Iran	2012-2014	Endoscopic	16	39.4 ± 15.21	N/S	9	N/S
				Microscopic	19	43.06 ± 11.29	N/S	10	N/S
Cho and Liau, 2002 [23]	Cohort	China	1996-2000	Endoscopic	22	45.3 (22-60)	22	0	42 (6-60)
				Microscopic	22	46.7 (18-56)	21	1	42 (6-60)
Messerer et al., 2011 [15]	Retrospective cohort	France	2006-2009	Endoscopic	82	57 (20-82)	35	47	12
				Microscopic	82	56.5 (27-84)	31	51	12
O'Malley et al., 2018 [24]	Retrospective cohort	USA	2003-2008	Endoscopic	25	47.8 (18-73)	10	15	4.91 (0.96-24.96)
				Microscopic	25	50.8 (23-78)	9	16	8.87 (0.96-42.96)
Kikuchi et al., 2017 [25]	Retrospective cohort	Japan	2005-2012	Endoscopic	21	56.2 (22-76)	8	13	N/S
				Microscopic	19	54 (27-72)	4	15	N/S
Zaidi et al., 2016 [26]	Retrospective cohort	USA	2011-2014	Endoscopic	55	55.9 ± 13.8	20	35	6
				Microscopic	80	59.1 ± 14.6	30	50	6
Levi et al., 2017 [27]	Retrospective cohort	Italy	2004-2012	Endoscopic	140	58.5	54	86	N/S
				Microscopic	81	52	37	44	N/S
Dallapiazza et al., 2014 [28]	Retrospective cohort	USA	2010-2013	Endoscopic	56	56.2 ± 12.8	29	27	12
				Microscopic	43	56.7 ± 16.9	19	24	12
Eseonu et al., 2017 [29]	Retrospective cohort	USA	2005-2015	Endoscopic	275	49 ± 16.2	N/S	115	N/S
				Microscopic	109	48.8 ± 15.8	N/S	48	N/S
Castaño-Leon et al., 2020 [30]	Retrospective cohort	Spain	1995-2017	Endoscopic	97	52	58	39	6
				Microscopic	90	45	54	36	6
Zhang et al., 2021 [31]	Randomized clinical trial	China	2017-2020	Endoscopic	23	55.6 (26-58)	10	13	N/S
				Microscopic	23	53.2 (23-60)	11	12	N/S
Vassilyeva et al., 2023 [32]	Randomized clinical trial	Bulgaria	2017-2021	Endoscopic	43	43.26 ± 8.64	26	17	6
				Microscopic	40	44.12 ± 9.14	24	16	6
Jain et al., 2007 [33]	Randomized clinical trial	India	N/S	Endoscopic	10	40.10	N/S	N/S	6.95 (3-15)
				Microscopic	10	31.6	N/S	N/S	6.95 (3-15)
Fan et al., 2025 [34]	Retrospective cohort	China	2015-2022	Endoscopic	48	46 ± 9	27	21	2
				Microscopic	55	43 ± 8	35	20	2
Ferat et al., 2026 [35]	Retrospective cohort	China	2009-2013	Endoscopic	40	46.2 ± 10.3	23	17	2
				Microscopic	31	46.2 ± 10.3	18	13	2
Kumar et al., 2025 [36]	Prospective cohort	India	N/S	Endoscopic	30	42.30 ± 10.25	14	16	N/S
				Microscopic	30	43.70 ± 11.12	13	17	N/S
Ordóñez-Rubiano et al., 2024 [37]	Retrospective cohort	Colombia	2018-2019	Endoscopic	18	50.2 ± 10.1	9	9	3
				Microscopic	11	52.3 ± 15.1	8	3	3
Savik et al., 2025 [38]	Retrospective cohort	Turkey	2012-2018	Endoscopic	26	50.6	N/S	N/S	2.7
				Microscopic	26	50.2	N/S	N/S	2.7
Zhang et al., 2025 [39]	Retrospective cohort	China	2011-2021	Endoscopic	1023	48 (37-57)	543	480	34
				Microscopic	840	47 (38-55)	421	419	34

The reporting of tumor characteristics was not uniform across the included studies and varied depending on the analyzed study (Table 2). Additionally, in some studies, tumor characteristics were not specified or were not well defined. Macroadenomas were more frequent, and the endoscopic technique was the most commonly used in these cases, followed by microadenomas, where the endoscopic technique was also the most frequently utilized. Regarding hormone secretion, non-functioning pituitary adenomas were the most common, followed in descending order by growth hormone tumors, adrenocorticotropic hormone (ACTH)-secreting tumors, prolactinomas, and thyroid-stimulating hormone-secreting tumors.

**Table 2:** Tumor characteristics ACTH, adrenocorticotropic hormone; GH, growth hormone; PRL, prolactin; TSH, thyroid-stimulating hormone; N/S, not specified

Study	Surgical Intervention	Number of Patients	Volume (cm^3^)	Classification by Size		Classification by Functionality					
						Nonfunctional Adenoma (%)	Functional Adenoma				
				Microadenoma (%)	Macroadenoma (%)		ACTH (%)	GH (%)	PRL (%)	TSH (%)	Others (%)
Van Gompel et al., 2021 [19]	Endoscopic	261	2.1 ± 0.1	N/S	N/S	116 (44)	58 (22)	38 (15)	17 (6.5)	4 (1.5)	28 (10.7)
	Microscopic	273	2 ± 0.1	N/S	N/S	137 (50)	54 (20)	45 (16)	11 (4)	3 (1.1)	23 (8.4)
Gao et al., 2016 [20]	Endoscopic	60	N/S	13	47	27	5	7	21	N/S	N/S
	Microscopic	45	N/S	10	35	22	3	4	16	N/S	N/S
Fathalla et al., 2015 [21]	Endoscopic	42	3.62	N/S	35 (83.3)	N/S	N/S	N/S	N/S	N/S	N/S
	Microscopic	23	2.70	N/S	17 (73.9)	N/S	N/S	N/S	N/S	N/S	N/S
Akbari et al., 2018 [22]	Endoscopic	16	3.63 ± 0.44	N/S	N/S	N/S	N/S	N/S	N/S	N/S	N/S
	Microscopic	19	3.4 ± 0.46	N/S	N/S	N/S	N/S	N/S	N/S	N/S	N/S
Cho and Liau, 2002 [23]	Endoscopic	22	N/S	11	11	N/S	N/S	N/S	22 (100)	N/S	N/S
	Microscopic	22	N/S	12	10	N/S	N/S	N/S	22 (100)	N/S	N/S
Messerer et al., 2011 [15]	Endoscopic	82	1.31	N/S	N/S	N/S	4 (4.9)	2 (2.4)	2 (2.4)	2 (2.4)	72 (87.8)
	Microscopic	82	1.45	N/S	N/S	N/S	2 (2.4)	4 (4.9)	1 (1.2)	0 (0)	75 (91.5)
O'Malley et al., 2018 [24]	Endoscopic	25	N/S	3 (12)	22 (8)	1	2	1	3	1	17
	Microscopic	25	N/S	2 (8)	23 (92)	7	1	1	0	0	16
Kikuchi et al., 2017 [25]	Endoscopic	21	8.4 (2.4-28.2)	N/S	N/S	21	N/S	N/S	N/S	N/S	N/S
	Microscopic	19	6.8 (1.2-21.8)	N/S	N/S	19	N/S	N/S	N/S	N/S	N/S
Zaidi et al., 2016 [26]	Endoscopic	55	13.4 ±14.5	N/S	N/S	55	N/S	N/S	N/S	N/S	N/S
	Microscopic	80	11.0 ± 12.4	N/S	N/S	80	N/S	N/S	N/S	N/S	N/S
Levi et al., 2017 [27]	Endoscopic	140	N/S	35 (25)	105 (75)	75 (54)	22	32	5	6	N/S
	Microscopic	81	N/S	16 (20)	65 (80)	44 (54)	15	14	5	3	N/S
Dallapiazza et al., 2014 [28]	Endoscopic	56	5.0	N/S	N/S	N/S	N/S	N/S	N/S	N/S	N/S
	Microscopic	43	5.6	N/S	N/S	N/S	N/S	N/S	N/S	N/S	N/S
Eseonu et al., 2017 [29]	Endoscopic	275	6.11 (6.7)	78 (28.4)	197 (71.6)	175 (63.6)	42 (15.3)	48 (17.5)	8 (2.9)	2 (0.73)	0
	Microscopic	109	5.75 (6.6)	28 (25.7)	81 (74.3)	74 (67.9)	14 (12.8)	15 (13.8)	2 (1.8)	3 (2.75)	1 (0.92)
Castaño-Leon et al., 2020 [30]	Endoscopic	97	N/S	N/S	80 (82.5)	N/S	19 (48.7)	16 (41)	4 (10.3)	0	0
	Microscopic	90	N/S	N/S	61 (67.8)	N/S	21 (42.9)	17 (34.7)	7 (14.6)	3 (6.1)	0
Zhang et al., 2021 [31]	Endoscopic	23	0.82 (0.5-2.5)	19 (83)	4 (17)	N/S	23	N/S	N/S	N/S	N/S
	Microscopic	23	0.89 (0.4-2.8)	18 (78)	5 (22)	N/S	23	N/S	N/S	N/S	N/S
Vassilyeva et al., 2023 [32]	Endoscopic	43	N/S	4 (9.3)	39 (90.7)	N/S	N/S	43	N/S	N/S	N/S
	Microscopic	40	N/S	3 (7.5)	37 (92.5)	N/S	N/S	40	N/S	N/S	N/S
Jain et al., 2007 [33]	Endoscopic	10	6.81	1	N/S	N/S	N/S	N/S	N/S	N/S	N/S
	Microscopic	10	4.80	2	N/S	N/S	N/S	N/S	N/S	N/S	N/S
Fan et al., 2025 [34]	Endoscopic	48	N/S	N/S	N/S	N/S	N/S	N/S	N/S	N/S	N/S
	Microscopic	55	N/S	N/S	N/S	N/S	N/S	N/S	N/S	N/S	N/S
Ferat et al., 2026 [35]	Endoscopic	40	N/S	36	64	42	N/S	N/S	N/S	N/S	N/S
	Microscopic	31	N/S	36	64	42	N/S	N/S	N/S	N/S	N/S
Kumar et al., 2025 [36]	Endoscopic	30	N/S	N/S	N/S	12 (40)	N/S	N/S	N/S	N/S	N/S
	Microscopic	30	N/S	N/S	N/S	13 (43)	N/S	N/S	N/S	N/S	N/S
Ordóñez-Rubiano et al., 2024 [37]	Endoscopic	18	9.6 ± 9.2	N/S	N/S	10 (55.6)	N/S	N/S	N/S	N/S	N/S
	Microscopic	11	2.1 ± 1	N/S	N/S	9 (81.8)	N/S	N/S	N/S	N/S	N/S
Savik et al., 2025 [38]	Endoscopic	26	N/S	N/S	N/S	10 (19.23)	6 (11.53)	8 (15.38)	2 (3.84)	N/S	N/S
	Microscopic	26	N/S	N/S	N/S	11 (21.15)	3 (5.76)	11 (21.15)	1 (1.92)	N/S	N/S
Zhang et al., 2025 [39]	Endoscopic	1023	2.6	11 (1.1)	872 (85.2)	N/S	27 (2.6)	184 (18.0)	168 (16.4)	11 (1.1)	N/S
	Microscopic	840	2.5	9 (1.1)	747 (88.9)	N/S	12 (1.4)	120 (14.3)	152 (18.1)	10 (1.2)	N/S

**Figure 2:**
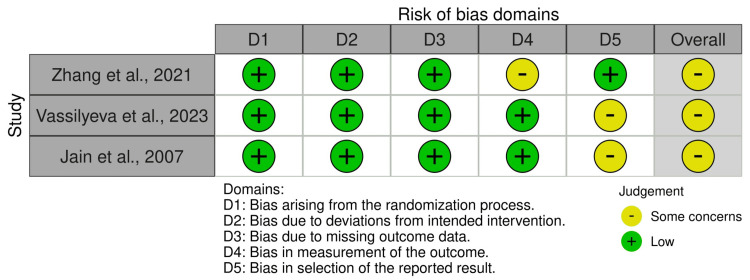
Evaluation of randomized clinical trials using the RoB 2 tool Note: Each domain was evaluated, resulting in a classification of low risk, some concerns, or high risk [16]. The overall risk of bias was determined based on the evaluation of the five domains, resulting in an overall rating of low risk, some concerns, or high risk. The RCTs evaluated are by Zhang et al. [31]​, Vassilyeva et al. [32]​, and Jain et al. [33]​ RCTs: randomized controlled trials

For the risk of bias assessment, the randomized clinical trials were assessed using the RoB 2 tool [17], while cohort studies were evaluated using the Newcastle-Ottawa Quality Assessment Scale [18]. Our included studies sum up to a total of 22. Three randomized clinical trials were identified, all of which were classified as "some concerns" (100%). For cohort studies, 19 studies were identified, of which 17 were classified as "good quality" (89.47%) and two as "fair quality" (10.52%). The details of the assessment for each study are presented in Table 3 and Figure 2.

**Table 3:** Newcastle-Ottawa tool for cohort studies' risk of bias appraisal Note: The Newcastle-Ottawa Quality Assessment Scale classifies studies as good quality, fair quality, or poor quality based on the number of stars obtained in each domain: good quality studies, 3-4 stars in selection, 1-2 in comparability, and 2-3 in outcome/exposure; fair quality studies: two stars in selection, 1-2 in comparability, and 2-3 in outcome/exposure; and poor quality studies: 0-1 star in selection, zero in comparability, and 0-1 in outcome/exposure [18]

Author and Year	Study Design	Selection	Comparability	Outcome/Exposure	Total	Subjective Evaluation
Van Gompel et al., 2021 [19]	Retrospective cohort	4	2	3	9	Good quality
Gao et al., 2016 [20]	Retrospective cohort	3	1	3	6	Fair quality
Fathalla et al., 2015 [21]	Retrospective cohort	4	1	3	8	Good quality
Akbari et al., 2018 [22]	Cohort	4	1	3	9	Good quality
Cho and Liau, 2002 [23]	Retrospective cohort	4	1	3	8	Good quality
Messerer et al., 2011 [15]	Retrospective cohort	4	2	3	9	Good quality
O'Malley et al., 2018 [24]	Retrospective cohort	4	2	3	9	Good quality
Kikuchi et al., 2017 [25]	Retrospective cohort	4	1	3	8	Good quality
Zaidi et al., 2016 [26]	Retrospective cohort	4	1	3	8	Good quality
Levi et al., 2017 [27]	Retrospective cohort	4	1	3	8	Good quality
Dallapiazza et al., 2014 [28]	Retrospective cohort	4	2	3	9	Good quality
Eseonu et al., 2017 [29]	Retrospective cohort	4	1	3	8	Good quality
Castaño-Leon et al., 2020 [30]	Retrospective cohort	4	2	3	9	Good quality
Fan et al., 2025 [34]	Retrospective cohort	4	1	2	7	Good quality
Ferat et al., 2026 [35]	Retrospective cohort	4	2	3	9	Good quality
Kumar et al., 2025 [36]	Prospective cohort	4	1	3	8	Good quality
Ordóñez-Rubiano et al., 2024 [37]	Retrospective cohort	4	1	2	7	Good quality
Savik et al., 2025 [38]	Retrospective cohort	4	1	1	6	Fair quality
Zhang et al., 2025 [39]	Retrospective cohort	4	2	3	9	Good quality

In Table 4 and Table 5, the postoperative results, as well as the complications for each of the surgical techniques, are described.

**Table 4:** Postoperative outcomes N/S, not specified; LOS, length of stay; PO, postoperative

Study	Surgical Intervention	Number of Patients	Resection		Operative Time (Minute) Mean ± SD (Range)	Hospital LOS (Day) Mean ± SD (Range)	Visual Field Improvement PO (%)
			Total (%)	Subtotal (%)			
Van Gompel et al., 2021 [19]	Endoscopic	261	N/S	N/S	131 ± 6	1.3 ± 0.05	N/S
	Microscopic	273	N/S	N/S	83 ± 7	1.3 ± 0.05	N/S
Gao et al., 2016 [20]	Endoscopic	60	49 (81.7)	11	175 ± 25	5.1 ± 0.7	21 (75)
	Microscopic	45	28 (62.2)	11	110 ± 17	7.8 ± 0.8	14 (70)
Fathalla et al., 2015 [21]	Endoscopic	42	25	15	N/S	4	N/S
	Microscopic	23	8	11	N/S	3.6	N/S
Akbari et al., 2018 [22]	Endoscopic	16	13	N/S	N/S	N/S	N/S
	Microscopic	19	3	N/S	N/S	N/S	N/S
Cho and Liau, 2002 [23]	Endoscopic	22	N/S	N/S	102 (60-180)	3.2 (2-5)	5 (63)
	Microscopic	22	N/S	N/S	162 (90-240)	5.3 (4-8)	6 (60)
Messerer et al., 2011 [15]	Endoscopic	82	61 (74)	21 (26)	N/S	N/S	29 (51)
	Microscopic	82	41 (50)	41 (50)	N/S	N/S	31 (50)
O'Malley et al., 2018 [24]	Endoscopic	25	14 (66)	N/S	176.4 (92.4-247.2)	3.92 (3-9)	N/S
	Microscopic	25	17 (77)	N/S	264.6 (175.8-525)	4.84 (3-9)	N/S
Kikuchi et al., 2017 [25]	Endoscopic	21	N/S	N/S	187 (147-308)	N/S	N/S
	Microscopic	19	N/S	N/S	184 (100-270)	N/S	N/S
Zaidi et al., 2016 [26]	Endoscopic	55	43 (78.2)	12	N/S	2.3 ± 1.2	N/S
	Microscopic	80	65 (81.3)	15	N/S	2.8 ± 3.4	N/S
Levi et al., 2017 [27]	Endoscopic	140	N/S	N/S	N/S	5	N/S
	Microscopic	81	N/S	N/S	N/S	7	N/S
Dallapiazza et al., 2014 [28]	Endoscopic	56	54 (96)	2	N/S	2.4 ± 0.76	N/S
	Microscopic	43	40 (93)	3	N/S	3 ± 1.7	N/S
Eseonu et al., 2017 [29]	Endoscopic	275	N/S	N/S	180.2	2.4 ± 1.2	100 (36.3)
	Microscopic	109	N/S	N/S	215.6	3.2 ± 2.2	42 (38.5)
Castaño-Leon et al., 2020 [30]	Endoscopic	97	62 (63.9)	N/S	150	6	25 (71.42)
	Microscopic	90	38 (42.2)	N/S	180	8	14 (46.7)
Zhang et al., 2021 [31]	Endoscopic	23	21	2	108 (60-192)	2.8 (2-5)	2 (67)
	Microscopic	23	20	3	174 (96-258)	5.1 (4-8)	3 (75)
Vassilyeva et al., 2023 [32]	Endoscopic	43	38 (88.4)	4 (9.3)	142 ± 54	5 ± 1.4	22
	Microscopic	40	27 (67.5)	10 (25)	176 ± 56	7 ± 1.4	10
Jain et al., 2007 [33]	Endoscopic	10	5	N/S	64.5 ± 19.16	N/S	10
	Microscopic	10	5	N/S	75.5 ± 18.48	N/S	10
Fan et al., 2025 [34]	Endoscopic	48	40 (83.3)	8 (16.7)	182 ± 11	8 (7-10)	11 (22.9)
	Microscopic	55	14 (25.5)	41 (74.5)	8 (7-10)	9 (8-11)	22 (40.0)
Ferat et al., 2026 [35]	Endoscopic	40	82	18	N/S	4.1 ± 1.2	70
	Microscopic	31	74	26	N/S	5.3 ± 1.6	58
Kumar et al., 2025 [36]	Endoscopic	30	26 (86.67)	4 (13.33)	N/S	N/S	25 (83.33)
	Microscopic	30	21 (70)	4 (13.33)	N/S	N/S	20 (66.67)
Ordóñez-Rubiano et al., 2024 [37]	Endoscopic	18	11 (61.1)	7 (38.9)	N/S	N/S	16 (88.9)
	Microscopic	11	6 (54.5)	5 (45.5)	N/S	N/S	8 (72.8)
Savik et al., 2025 [38]	Endoscopic	26	17 (32.69)	9 (17.30)	N/S	N/S	81.8
	Microscopic	26	17 (32.69)	7 (13.46)	N/S	N/S	88.9
Zhang et al., 2025 [39]	Endoscopic	1023	490 (59.5)	323 (39.2)	120 (84-160)	8.0 (6.0-12)	N/S
	Microscopic	840	447 (54.3)	367 (44.6)	84 (60-100)	8.4 (7.0-11)	N/S

**Table 5:** Postoperative complications PO, postoperative; N/S, not specified; CSF, cerebrospinal fluid

Study	Surgical Intervention	Number of Patients	Number of Cases (%)							
			Visual Field Deterioration PO	CSF Leak PO	Diabetes Insipidus PO (Transient and Permanent)	Hypopituitarism PO	Sinus Infection PO	Epistaxis PO	Meningitis PO	Death
Van Gompel et al., 2021 [19]	Endoscopic	261	0	5 (1.9)	5 (1.9)	19 (7.3)	N/S	N/S	N/S	0
	Microscopic	273	2 (0.7)	6 (2.2)	10 (3.7)	23 (8.4)	N/S	N/S	N/S	1 (0.4)
Gao et al., 2016 [20]	Endoscopic	60	N/S	6	2	2	N/S	1	0	0
	Microscopic	45	N/S	5	1	7	N/S	2	1	0
Fathalla et al., 2015 [21]	Endoscopic	42	N/S	2 (4.7)	9 (21.8)	5 (11.9)	4 (9.5)	1 (2.3)	N/S	N/S
	Microscopic	23	N/S	2 (8.6)	12 (52)	1 (4.3)	2 (8.6)	1 (4.3)	N/S	N/S
Akbari et al., 2018 [22]	Endoscopic	16	5 (31.2)	3 (18.8)	5 (31.2)	12 (75)	N/S	N/S	2 (12.5)	2 (12.5)
	Microscopic	19	5 (26.3)	2 (10.5)	4 (21)	11 (57.8)	N/S	N/S	1 (5.2)	2 (12.5)
Cho and Liau, 2002 [23]	Endoscopic	22	N/S	0	N/S	0	1	0	N/S	N/S
	Microscopic	22	N/S	1	N/S	1	2	1	N/S	N/S
Messerer et al., 2011 [15]	Endoscopic	82	0	N/S	7 (8.5)	5	0	4 (4.9)	3 (3.7)	0
	Microscopic	82	1 (2)	N/S	8 (9.8)	9	0	1 (1.2)	4 (4.9)	0
O'Malley et al., 2018 [24]	Endoscopic	25	1	3 (12)	1 (4)	0	0	0	0	0
	Microscopic	25	1	1 (4)	6 (24)	1	0	0	1	0
Kikuchi et al., 2017 [25]	Endoscopic	21	13	0	1	1	0	1	0	0
	Microscopic	19	10	0	N/S	0	0	0	0	0
Zaidi et al., 2016 [26]	Endoscopic	55	0	0	1 (1.8)	N/S	N/S	0	0	N/S
	Microscopic	80	0	2 (2.5)	7 (8.8)	N/S	N/S	1 (1.3)	0	N/S
Levi et al., 2017 [27]	Endoscopic	140	N/S	N/S	3 (2.1)	N/S	N/S	2 (1.4)	0	N/S
	Microscopic	81	N/S	N/S	3 (3.7)	N/S	N/S	0	0	N/S
Dallapiazza et al., 2014 [28]	Endoscopic	56	N/S	4 (7)	10	N/S	5	6	N/S	N/S
	Microscopic	43	N/S	5 (12)	7	N/S	1	1	1	N/S
Eseonu et al., 2017 [29]	Endoscopic	275	15 (5.49)	10 (3.6)	2 (0.7)	9 (3.3)	1	1 (0.36)	2 (0.73)	N/S
	Microscopic	109	4 (3.7)	8 (7.3)	1 (0.9)	7 (6.4)	0	0	0	N/S
Castaño-Leon et al., 2020 [30]	Endoscopic	97	N/S	2 (2.1)	29	N/S	N/S	8 (8.2)	4 (4.1)	0
	Microscopic	90	N/S	8 (8.9)	38	N/S	N/S	7 (7.8)	2 (2.2)	0
Zhang et al., 2021 [31]	Endoscopic	23	N/S	0	N/S	0	2	1	N/S	N/S
	Microscopic	23	N/S	1	N/S	2	2	1	N/S	N/S
Vassilyeva et al., 2023 [32]	Endoscopic	43	N/S	N/S	8 (18.6)	N/S	0	2	0	0
	Microscopic	40	N/S	N/S	6 (15)	N/S	0	0	0	0
Jain et al., 2007 [33]	Endoscopic	10	0	1	1	6	0	N/S	N/S	N/S
	Microscopic	10	0	1	2	7	2	N/S	N/S	N/S
Fan et al., 2025 [34]	Endoscopic	48	N/S	8 (16.7)	4 (8.3)	9 (18.8)	N/S	N/S	N/S	N/S
	Microscopic	55	N/S	2 (3.6)	2 (3.6)	6 (10.9)	N/S	N/S	N/S	N/S
Ferat et al., 2026 [35]	Endoscopic	40	0	5	12	10	N/S	N/S	0	0
	Microscopic	31	0	6	15	13	N/S	N/S	3.2	0
Kumar et al., 2025 [36]	Endoscopic	30	2 (6.67)	3 (10.00)	2 (6.67)	N/S	N/S	N/S	1 (3.33)	0
	Microscopic	30	4 (13.33)	5 (16.67)	3 (10.00)	N/S	N/S	N/S	2 (6.67)	0
Ordóñez-Rubiano et al., 2024 [37]	Endoscopic	18	2 (11.1)	1 (5.5)	1 (5.5%)	N/S	N/S	N/S	N/S	0
	Microscopic	11	3 (27.2)	1 (9.1)	1 (9.1%)	N/S	N/S	N/S	N/S	0
Savik et al., 2025 [38]	Endoscopic	26	N/S	3	9	N/S	N/S	N/S	N/S	0
	Microscopic	26	N/S	2	5	N/S	N/S	N/S	N/S	0
Zhang et al., 2025 [39]	Endoscopic	1023	N/S	183 (22.2)	N/S	N/S	N/S	N/S	N/S	N/S
	Microscopic	840	N/S	156 (19.0)	N/S	N/S	N/S	N/S	N/S	N/S

Results of Individual Studies

A total of 22 studies were included, comprising 4390 patients, of which 2413 underwent ETS and 1973 MTS. Most of the studies were retrospective cohort studies, with a smaller proportion of randomized controlled trials. The participants were between 18 and 84 years old, and the studies were conducted in various countries. The number of participants per study ranged from 10 to 1023. Macroadenomas were reported more frequently than microadenomas, and non-functional adenomas were the most common tumor subtype.

Surgical Outcomes

GTR rates were greater for the ETS than for the MTS in all included trials. Resection rates for ETS and MTS were 91.7% and 86.9%, respectively, according to Van Gompel et al. (2021) [19]. In a similar vein, Castaño-Leon et al. (2020) reported GTR rates of 42.2% in the MTS group and 63.9% in the ETS group (p < 0.001) [30]. While Zhang et al. (2025) [39] discovered greater GTR rates with ETS following propensity score matching (59.5% versus 54.3%, p = 0.037), Ferat et al. (2026) [35] likewise observed higher GTR rates with ETS (82% versus 74%, p = 0.04).

Each study had a different operational time. According to Van Gompel et al. (2021), the MTS group had a shorter operating time (83 ± 7 minutes) than the ETS group (131 ± 6 minutes, p < 0.001) [19]. On the other hand, Eseonu et al. (2017) [29] and Vassilyeva et al. (2023) [32] found that the ETS group had lower operating times than the MTS group.

The ETS group consistently had reduced LOS. An average LOS of five days for ETS and seven days for MTS was reported by Levi et al. (2017) [27]. While Vassilyeva et al. (2023) [32] found 5 ± 1.4 days for ETS and 7 ± 1.4 days for MTS (p < 0.001), Eseonu et al. (2017) [29] observed LOS of 2.4 days for ETS and 3.2 days for MTS (p = 0.03). Additionally, Ferat et al. (2026) found that the endoscopic group had a lower length of stay (4.1 ± 1.2 versus 5.3 ± 1.6 days, p = 0.01) [35].

Visual outcomes were reported in multiple studies. Vassilyeva et al. (2023) [32] reported visual improvement in 51.8% of patients undergoing ETS compared to 25.9% in the MTS group (p < 0.05), while Ferat et al. (2026) [35] reported improvement in 70% and 58% of cases, respectively (p = 0.03). Ordóñez-Rubiano et al. (2024) reported stable or improved vision in 88.9% of ETS patients compared to 72.8% in MTS, although this difference was not statistically significant (p = 0.147) [37]. Other studies reported no significant differences in visual outcomes between techniques.

Postoperative Complications

Postoperative complication rates were comparable between ETS and MTS across studies. In addition to comparable rates of hypopituitarism and diabetes insipidus, Van Gompel et al. (2021) revealed CSF leak rates of 1.9% in the ETS group and 2.2% in the MTS group (p = 0.8) [19]. Additionally, Levi et al. (2017) found no discernible variations in vision impairment, diabetes insipidus, or CSF leak between methods [27].

Eseonu et al. (2017) reported CSF leak rates of 3.1% in the ETS group and 7.3% in the MTS group, with similar rates of hypopituitarism and no differences in visual outcomes [29]. Castaño-Leon et al. (2020) reported differences in temporary diabetes insipidus and CSF leak between techniques, while permanent diabetes insipidus and meningitis rates were similar [30].

Zhang et al. (2021) reported overall complication rates of 34.78% in the ETS group and 13.04% in the MTS group [31]. Vassilyeva et al. (2023) reported mostly mild complications, with no cases of meningitis or mortality in either group [32]. Ferat et al. (2026) reported similar rates of CSF leak (5% versus 6%), diabetes insipidus (12% versus 15%), and hypopituitarism (10% versus 13%), with no perioperative mortality [35].

Ordóñez-Rubiano et al. (2024) reported low complication rates, including a CSF leak rate of 2.9%, with no significant differences between techniques [37]. Zhang et al. (2025) reported no significant differences in major complications, including CSF leak, vascular injury, or reoperation [39].

Discussion

In the context of the current surgical management of pituitary adenomas, ongoing debate persists regarding the relative advantages of endoscopic and microscopic transsphenoidal techniques. The transsphenoidal approach has become the gold standard for the treatment of this condition, with two main techniques being used: microscopic and endoscopic transsphenoidal surgery [9]. Although there is literature comparing both techniques, it remains a subject of ongoing analysis regarding differences in complications, recovery times, and total tumor resection [11]. This systematic review contributes to this debate by emphasizing the degree of tumor resection, complications, and recovery times between endoscopic and microscopic transsphenoidal techniques as treatment for pituitary adenomas.

The data analysis of 4390 participants from different regions around the world provides a comprehensive evaluation of the outcomes in the comparison between the ETS and MTS techniques for the treatment of pituitary adenomas. Our results indicate that, on average, GTR is achieved more in patients with the endoscopic technique (76%) compared to the microscopic technique (60.8%). This difference is likely related to the enhanced visualization provided by the endoscopic approach, including a wider field of view and improved illumination, which facilitates a better identification of tumor margins and access to complex anatomical regions such as suprasellar and parasellar extensions. This higher GTR rate shows how significant endoscopic surgery is for getting a bigger tumor resection, which is an important surgical endpoint in the treatment of pituitary adenomas [24]. Beyond GTR, maintaining pituitary function is a crucial factor in surgical success, especially in functional adenomas where the danger of endocrine deficits following surgery must be weighed against the possibility of establishing hormonal remission. Hormonal remission rates in our sample were similar between endoscopic and microscopic methods (65% versus 62%), and there were no discernible differences between methods for transient diabetes insipidus (12% versus 15%) and new-onset hypopituitarism (10% versus 13%) [36]. These results are in line with other research showing that tumor biology, the degree of resection, and surgical pituitary gland manipulation had a greater impact on endocrine outcomes than the imaging modality alone [37].

Postoperative complication rates varied significantly across the studies. Reported cerebrospinal fluid leak rates ranged from 0% to 18.8% in endoscopic series and from 0% to 10.5% in microscopic series. Postoperative diabetes insipidus occurred from 0% to 21.8% of cases following endoscopic surgery and from 0% to 24% following microscopic surgery. Hypopituitarism showed wide and overlapping ranges between both techniques, with rates from 0% to 75% in endoscopic series and from 0% to 57.8% in microscopic series. In contrast, postoperative epistaxis was reported slightly more often in endoscopic series, ranging from 0% to 8.2%, compared to 0% to 7.8% in microscopic series. However, it was demonstrated that endoscopic surgery for pituitary adenomas is associated with low mortality. While the endoscopic transsphenoidal approach achieved higher rates of gross total resection, postoperative complication rates were variable and showed substantial overlap between endoscopic and microscopic techniques. Although the endoscopic technique may provide some benefits, overall safety and recovery outcomes seem to be affected more by study features and the surgeon's experience than by the surgical method itself.

Despite the benefits of the endoscopic transsphenoidal technique, its implementation in clinical practice faces several challenges. Factors such as the learning curve associated with the transition from microscopic to endoscopic transsphenoidal surgery may influence early surgical outcomes. However, evidence from comparative studies and large surgical series indicates that the rates of complications and clinical outcomes are similar to those observed in high-volume centers as surgical experience grows and endoscopic training is incorporated into standard neurosurgical practice [9,11,22,24].

Our findings align with previous studies, such as the systematic review and meta-analysis by Li et al. (2017), which included 23 studies assessing a total of 2272 patients with pituitary adenomas. This study emphasizes that the use of the ETS was associated with a higher incidence of GTR (OR, 1.52; 95% CI, 1.11-2.08; P = 0.009) compared to the MTS. Furthermore, ETS was associated with a reduction in the risk of diabetes insipidus and was not associated with the risk of meningitis, hypopituitarism, or mortality [40]. Several included studies reported similar results. Messerer et al. (2011) found that the quality of resection after one year of surgery was significantly higher in the endoscopic technique (GTR: 74% versus 50% in the microscopic approach; p = 0.002) [15]. Vassilyeva et al. (2023) evaluated patients with acromegaly, where the etiology in all cases was somatotropinomas, finding that complete tumor resection was 1.4 times more frequent (p < 0.05) with the endoscopic technique compared to the microscopic technique [32]. An important finding to mention is that reported by Van Gompel et al. (2021) regarding the operative time, where, in the endoscopic group, it was 131 ± 6 minutes, while in the microscopic group, it was 83 ± 7 minutes, making it 48 minutes shorter in the latter [19]. This may be due to the fact that no more tumors can be observed with the margins provided by the microscopic technique.

Some limitations in the study should be mentioned. Some of the studies included in this review may not have had a sufficient follow-up period to assess the safety and efficacy of the procedures. Other limitations include the heterogeneity of the data among the included studies and the lack of uniformity in data reporting, which negatively impacts the robustness of the conclusions. This literature is also limited by the retrospective design of many of the included articles. Although three randomized clinical trials are included, the methodological quality of these studies raises some concerns. The strengths of the study include the methodological quality of the included studies, where most of the retrospective studies are of good quality. There is an adequate sample size of the population undergoing ETS and MTS. Additionally, the included articles provide a specific conclusion.

Despite the relatively large sample size, heterogeneity across the included studies limits the ability to draw definitive conclusions for each evaluated outcome. These findings highlight the need for randomized controlled trials comparing both techniques, with standardized methodologies and adequate follow-up periods to better assess safety and clinical outcomes.

## Conclusions

Our study evaluates the efficacy, safety, and outcomes of the ETS and MTS as treatment options for pituitary adenomas. While the endoscopic approach may offer advantages in surgical visualization and tumor removal, clinically meaningful outcomes, including complication rates, recovery, and the preservation of pituitary function, remain comparable between techniques.

Importantly, maintaining pituitary function is still a vital goal in the surgical treatment of pituitary adenomas, especially in functioning tumors. Endocrine outcomes, such as hypopituitarism and diabetes insipidus, were similar across procedures in this research, underscoring the need for surgical success to strike a balance between tumor excision and maintaining normal gland function. These findings support an individualized approach to surgical decision-making, where surgeon expertise, tumor characteristics, and intraoperative strategy play a central role. Future research should focus on better defining these factors through standardized and stratified analyses, allowing for more precise and patient-centered treatment selection.

## Appendices

Search strategy

PubMed

The search terms used are the following: "pituitary neoplasms"[MeSH Terms] AND ((("microscop"[All Fields] OR "microscopal"[All Fields] OR "microscope"[All Fields] OR "microscope s"[All Fields] OR "microscopes"[All Fields] OR "microscopic"[All Fields] OR "microscopical"[All Fields] OR "microscopically"[All Fields] OR "microscopics"[All Fields]) AND ("transsphenoid"[All Fields] OR "transsphenoidal"[All Fields] OR "transsphenoidally"[All Fields]) AND ("approach"[All Fields] OR "approach s"[All Fields] OR "approachability"[All Fields] OR "approachable"[All Fields] OR "approache"[All Fields] OR "approached"[All Fields] OR "approaches"[All Fields] OR "approaching"[All Fields] OR "approachs"[All Fields])) OR (("microscop"[All Fields] OR "microscopal"[All Fields] OR "microscope"[All Fields] OR "microscope s"[All Fields] OR "microscopes"[All Fields] OR "microscopic"[All Fields] OR "microscopical"[All Fields] OR "microscopically"[All Fields] OR "microscopics"[All Fields]) AND ("pituitaries"[All Fields] OR "pituitary gland"[MeSH Terms] OR ("pituitary"[All Fields] AND "gland"[All Fields]) OR "pituitary gland"[All Fields] OR "pituitary"[All Fields] OR "pituitary s"[All Fields]) AND ("surgery"[MeSH Subheading] OR "surgery"[All Fields] OR "surgical procedures, operative"[MeSH Terms] OR ("surgical"[All Fields] AND "procedures"[All Fields] AND "operative"[All Fields]) OR "operative surgical procedures"[All Fields] OR "general surgery"[MeSH Terms] OR ("general"[All Fields] AND "surgery"[All Fields]) OR "general surgery"[All Fields] OR "surgery s"[All Fields] OR "surgerys"[All Fields] OR "surgeries"[All Fields])) OR (("microsurgical"[All Fields] OR "microsurgically"[All Fields]) AND ("transsphenoid"[All Fields] OR "transsphenoidal"[All Fields] OR "transsphenoidally"[All Fields]) AND ("methods"[MeSH Terms] OR "methods"[All Fields] OR "procedure"[All Fields] OR "methods"[MeSH Subheading] OR "procedures"[All Fields] OR "procedural"[All Fields] OR "procedurally"[All Fields] OR "procedure s"[All Fields])) OR (("transsphenoid"[All Fields] OR "transsphenoidal"[All Fields] OR "transsphenoidally"[All Fields]) AND ("microsurgery"[MeSH Terms] OR "microsurgery"[All Fields] OR "microsurgeries"[All Fields]))) AND ((("endoscope s"[All Fields] OR "endoscoped"[All Fields] OR "endoscopes"[MeSH Terms] OR "endoscopes"[All Fields] OR "endoscope"[All Fields] OR "endoscopical"[All Fields] OR "endoscopically"[All Fields] OR "endoscopy"[MeSH Terms] OR "endoscopy"[All Fields] OR "endoscopic"[All Fields]) AND ("pituitaries"[All Fields] OR "pituitary gland"[MeSH Terms] OR ("pituitary"[All Fields] AND "gland"[All Fields]) OR "pituitary gland"[All Fields] OR "pituitary"[All Fields] OR "pituitary s"[All Fields]) AND ("surgery"[MeSH Subheading] OR "surgery"[All Fields] OR "surgical procedures, operative"[MeSH Terms] OR ("surgical"[All Fields] AND "procedures"[All Fields] AND "operative"[All Fields]) OR "operative surgical procedures"[All Fields] OR "general surgery"[MeSH Terms] OR ("general"[All Fields] AND "surgery"[All Fields]) OR "general surgery"[All Fields] OR "surgery s"[All Fields] OR "surgerys"[All Fields] OR "surgeries"[All Fields])) OR (("endonasal"[All Fields] OR "endonasally"[All Fields]) AND ("transsphenoid"[All Fields] OR "transsphenoidal"[All Fields] OR "transsphenoidally"[All Fields]) AND ("surgery"[MeSH Subheading] OR "surgery"[All Fields] OR "surgical procedures, operative"[MeSH Terms] OR ("surgical"[All Fields] AND "procedures"[All Fields] AND "operative"[All Fields]) OR "operative surgical procedures"[All Fields] OR "general surgery"[MeSH Terms] OR ("general"[All Fields] AND "surgery"[All Fields]) OR "general surgery"[All Fields] OR "surgery s"[All Fields] OR "surgerys"[All Fields] OR "surgeries"[All Fields])) OR (("endoscope s"[All Fields] OR "endoscoped"[All Fields] OR "endoscopes"[MeSH Terms] OR "endoscopes"[All Fields] OR "endoscope"[All Fields] OR "endoscopical"[All Fields] OR "endoscopically"[All Fields] OR "endoscopy"[MeSH Terms] OR "endoscopy"[All Fields] OR "endoscopic"[All Fields]) AND ("transsphenoid"[All Fields] OR "transsphenoidal"[All Fields] OR "transsphenoidally"[All Fields]) AND ("resect"[All Fields] OR "resectability"[All Fields] OR "resectable"[All Fields] OR "resectates"[All Fields] OR "resected"[All Fields] OR "resecting"[All Fields] OR "resection"[All Fields] OR "resectional"[All Fields] OR "resectioned"[All Fields] OR "resectioning"[All Fields] OR "resections"[All Fields] OR "resective"[All Fields] OR "resects"[All Fields])) OR (("transsphenoid"[All Fields] OR "transsphenoidal"[All Fields] OR "transsphenoidally"[All Fields]) AND ("endoscope s"[All Fields] OR "endoscoped"[All Fields] OR "endoscopes"[MeSH Terms] OR "endoscopes"[All Fields] OR "endoscope"[All Fields] OR "endoscopical"[All Fields] OR "endoscopically"[All Fields] OR "endoscopy"[MeSH Terms] OR "endoscopy"[All Fields] OR "endoscopic"[All Fields]) AND ("microsurgery"[MeSH Terms] OR "microsurgery"[All Fields] OR "microsurgeries"[All Fields]))).

## References

[1] Hong GK, Payne SC, Jane JA Jr. (2016). Anatomy, physiology, and laboratory evaluation of the pituitary gland. Otolaryngol Clin North Am.

[2] (2024). Chapter 18: the pituitary gland. Ganong’s Medical Physiology Examination & Board Review.

[3] Lake MG, Krook LS, Cruz SV (2013). Pituitary adenomas: an overview. Am Fam Physician.

[4] Tritos NA, Miller KK (2023). Diagnosis and management of pituitary adenomas: a review. JAMA.

[5] Jane JA Jr., Catalino MP, Edward ER Jr. (2022). Surgical Treatment of Pituitary Adenomas.

[6] (2024). Pituitary adenoma. https://www.clinicalkey.com/#!/content/derived_clinical_overview/76-s2.0-B978032375576400716X.

[7] Würth R, Thellung S, Corsaro A, Barbieri F, Florio T (2020). Experimental evidence and clinical implications of pituitary adenoma stem cells. Front Endocrinol.

[8] Melmed S, Kaiser UB, Lopes MB (2022). Clinical biology of the pituitary adenoma. Endocr Rev.

[9] Esposito D, Olsson DS, Ragnarsson O, Buchfelder M, Skoglund T, Johannsson G (2019). Non-functioning pituitary adenomas: indications for pituitary surgery and post-surgical management. Pituitary.

[10] Swearingen B (2026). Transsphenoidal surgery for pituitary adenomas and other sellar masses. UpToDate.

[11] Guinto G, Guinto-Nishimura GY, Sangrador-Deitos MV (2023). Current and future perspectives of microscopic and endoscopic transsphenoidal surgery for pituitary adenomas: a narrative review. Arch Med Res.

[12] Martinez EC, Flores Valdés JR, Castillo JL (2023). Ten steps to conduct a systematic review. Cureus.

[13] Page MJ, McKenzie JE, Bossuyt PM (2021). The PRISMA 2020 statement: an updated guideline for reporting systematic reviews. BMJ.

[14] World Medical Association (2013). World Medical Association Declaration of Helsinki: ethical principles for medical research involving human subjects. JAMA.

[15] Messerer M, De Battista JC, Raverot G (2011). Evidence of improved surgical outcome following endoscopy for nonfunctioning pituitary adenoma removal. Neurosurg Focus.

[16] Ouzzani M, Hammady H, Fedorowicz Z, Elmagarmid A (2016). Rayyan-a web and mobile app for systematic reviews. Syst Rev.

[17] Sterne JA, Savović J, Page MJ (2019). RoB 2: a revised tool for assessing risk of bias in randomised trials. BMJ.

[18] Lo CK, Mertz D, Loeb M (2014). Newcastle-Ottawa Scale: comparing reviewers' to authors' assessments. BMC Med Res Methodol.

[19] Van Gompel JJ, Atkinson JL, Choby G (2021). Pituitary tumor surgery: comparison of endoscopic and microscopic techniques at a single center. Mayo Clin Proc.

[20] Gao Y, Zheng H, Xu S, Zheng Y, Wang Y, Jiang J, Zhong C (2016). Endoscopic versus microscopic approach in pituitary surgery. J Craniofac Surg.

[21] Fathalla H, Cusimano MD, Di Ieva A (2015). Endoscopic versus microscopic approach for surgical treatment of acromegaly. Neurosurg Rev.

[22] Akbari H, Malek M, Ghorbani M (2018). Clinical outcomes of endoscopic versus microscopic trans-sphenoidal surgery for large pituitary adenoma. Br J Neurosurg.

[23] Cho DY, Liau WR (2002). Comparison of endonasal endoscopic surgery and sublabial microsurgery for prolactinomas. Surg Neurol.

[24] O'Malley BW Jr, Grady MS, Gabel BC (2008). Comparison of endoscopic and microscopic removal of pituitary adenomas: single-surgeon experience and the learning curve. Neurosurg Focus.

[25] Kikuchi R, Toda M, Tomita T, Ogawa K, Yoshida K (2017). Surgical outcome of endoscopic endonasal surgery for non-functional pituitary adenoma by a team of neurosurgeons and otolaryngologists adenoma by a team of neurosurgeons and otolaryngologists. Turk Neurosurg.

[26] Zaidi HA, Awad AW, Bohl MA (2016). Comparison of outcomes between a less experienced surgeon using a fully endoscopic technique and a very experienced surgeon using a microscopic transsphenoidal technique for pituitary adenoma. J Neurosurg.

[27] Levi V, Bertani GA, Guastella C (2017). Microscopic versus endoscopic transsphenoidal surgery for pituitary adenoma: analysis of surgical safety in 221 consecutive patients. Clin Otolaryngol.

[28] Dallapiazza R, Bond AE, Grober Y, Louis RG, Payne SC, Oldfield EH, Jane JA Jr (2014). Retrospective analysis of a concurrent series of microscopic versus endoscopic transsphenoidal surgeries for Knosp grades 0-2 nonfunctioning pituitary macroadenomas at a single institution. J Neurosurg.

[29] Eseonu CI, ReFaey K, Rincon-Torroella J, Garcia O, Wand GS, Salvatori R, Quinones-Hinojosa A (2017). Endoscopic versus microscopic transsphenoidal approach for pituitary adenomas: comparison of outcomes during the transition of methods of a single surgeon. World Neurosurg.

[30] Castaño-Leon AM, Paredes I, Munarriz PM (2020). Endoscopic transnasal trans-sphenoidal approach for pituitary adenomas: a comparison to the microscopic approach cohort by propensity score analysis. Neurosurgery.

[31] Zhang T, Zhang B, Yuan L, Song Y, Wang F (2021). Superiority of endoscopic transsphenoidal pituitary surgery to microscopic transseptal pituitary surgery for treatment of Cushing's disease. Rev Assoc Med Bras (1992).

[32] Vassilyeva N, Mena N, Kirov K, Diatlova E (2023). Comparative effectiveness of endoscopic and microscopic adenoma removal in acromegaly. Front Endocrinol (Lausanne).

[33] Jain AK, Gupta AK, Pathak A, Bhansali A, Bapuraj JR (2007). Excision of pituitary adenomas: randomized comparison of surgical modalities. Br J Neurosurg.

[34] Fan R, Zhao R, Zhong Y, Wan W (2025). Long-term quality of life after microscopic and endoscopic transsphenoidal pituitary adenoma surgery: a retrospective cohort study. Asian J Surg.

[35] Ferat M, Müderris T, Ayberk G, Türkoğlu ÖF (2026). Comparative analysis of postoperative outcomes between endoscopic and microscopic transsphenoidal surgery for pituitary adenomas. Med Records.

[36] Kumar A, Saxena S, Lalchandani T (2025). Prospective assessment of endoscopic versus microscopic transsphenoidal pituitary surgery: functional outcomes and recurrence rates. Int J Life Sci Biotechnol Pharma Res.

[37] Ordóñez-Rubiano EG, Capacho-Delgado YA, Jacomussi-Alzate L (2024). Shaping the curve from the microscopic transsphenoidal to the endoscopic endonasal approach for the sellar region. Cir Cir.

[38] Savik R, Ozturk Y, Yangi K, Bozkurt I, Kalayci M (2025). Comparison of microscopic and endoscopic transsphenoidal surgery for pituitary adenomas. Cureus.

[39] Zhang C, Ma S, Xiao D (2025). Comparison of endoscopic versus microscopic transsphenoidal surgery in patients with pituitary adenomas: a propensity score matched study. Int J Surg.

[40] Li A, Liu W, Cao P, Zheng Y, Bu Z, Zhou T (2017). Endoscopic versus microscopic transsphenoidal surgery in the treatment of pituitary adenoma: a systematic review and meta-analysis. World Neurosurg.

